# Correction: Valenzuela-Reyes et al. Enhancing 3D Printing of Gelatin/Siloxane-Based Cellular Scaffolds Using a Computational Model. *Polymers* 2025, *17*, 1838

**DOI:** 10.3390/polym18161976

**Published:** 2026-08-14

**Authors:** Marcos B. Valenzuela-Reyes, Esmeralda S. Zuñiga-Aguilar, Christian Chapa-González, Javier S. Castro-Carmona, Luis C. Méndez-González, R. Álvarez-López, Humberto Monreal-Romero, Carlos A. Martínez-Pérez

**Affiliations:** 1 Institute of Engineering and Technology, Autonomous University of the City of Juarez, Ciudad Juárez 32310, Mexico; marcos.valenzuela@uacj.mx (M.B.V.-R.); esmeralda.zuniga@uacj.mx (E.S.Z.-A.); christian.chapa@uacj.mx (C.C.-G.); jcastro@uacj.mx (J.S.C.-C.); luis.mendez@uacj.mx (L.C.M.-G.); 2 Computer Science Department, The University of Texas at El Paso, West University Avenue, El Paso, TX 79968, USA; ralvarezlo1@miners.utep.edu; 3 Department of Biomaterials and Nanotechnology, Autonomous University of Chihuahua, University Av., Col. Centro 31000, Mexico; hmonreal@uach.mx

There was an error in the original publication [1].

The original publication contained wording that could be interpreted as overstating optimization, superiority, synergistic effects, high-fidelity three-dimensional printing, and biological scope beyond the direct evidence provided. In addition, the manuscript required clarification that the reported similarity values were derived from 2D image-based metrics and therefore should be interpreted as comparative proxies for planar CAD agreement under the tested conditions, rather than direct measures of full 3D fidelity, generalized process optimization, material superiority, or biological performance. To address the Editorial Board comments, residual wording such as “optimize”, “optimal”, “ideal”, “high-fidelity”, “accurate approach”, “rigorous validation”, “best structural and physicochemical performance”, and direct comparison of AUC and R^2^ as equivalent metrics have been revised throughout the manuscript.

**A correction has been made to Abstract, Paragraph 1:**

In recent years, extrusion-based 3D printing has been explored for the fabrication of hydrogel-based structures. This work presents an evaluation of the printability of a gelatin/siloxane hybrid material under defined extrusion conditions by combining rheological characterization, finite-element-based flow simulation in SolidWorks, and image-based analysis of printed meshes. Rheological tests revealed a working region around 50 Pa shear stress, with a maximum viscosity of 3 × 10^5^ Pa·s, a minimum viscosity of 0.089 Pa·s, and a shear rate of 15 s^−1^. Initial printing conditions using a 27G nozzle, 180 kPa pressure, 32 °C temperature, and 30 mm/s velocity produced mesh images with 54.5% similarity to the CAD target. A FEM-guided parameter set and a binary convolutional neural network trained on microscope images were then used as complementary screening tools to compare printed meshes under the tested conditions. Prints obtained with a 25G nozzle, 170 kPa pressure, 32 °C temperature, and 25 mm/s velocity reached 92.35% image-derived similarity to the CAD target, while the highest similarity observed among the evaluated mesh images was 94.13%. These results support the use of the proposed workflow as a comparative screening strategy for planar printability and CAD similarity in this gelatin/siloxane system. However, the similarity values reported here are derived from 2D image-based metrics and should not be interpreted as direct evidence of full 3D fidelity, generalized process optimization, material superiority, or biological performance.

**A correction has been made to Section 1. Introduction, Paragraphs 1–6:**

Bioinks are formulations composed of biomaterials and, in some cases, living cells or other supporting components used in extrusion-based three-dimensional printing to fabricate hydrogel-based structures. These materials are designed to provide suitable physicochemical and mechanical conditions for cell-related or tissue-engineering applications, including adhesion, growth, and differentiation, depending on their composition and intended use. Bioinks can include hydrogels, microcarriers, cellular aggregates, and decellularized matrices, each presenting specific material and processing characteristics [1–3].

Hybrid bioinks that integrate multiple biomaterials may offer complementary mechanical, structural, and biological properties compared with single-component systems [4]. This approach has been explored for the fabrication of hydrogel-based constructs and tissue-engineering models [5]. Examples of hybrid bioinks include hydrogel–ceramic composites for tissue engineering [6–8], hydrogel–microcarrier systems for organ printing [9], hydrogel–polymer systems for soft tissues, and hydrogel–bioactive material combinations to support cell adhesion, proliferation, and differentiation [10–12]. For instance, hydrogel–microcarrier hybrids have shown promise in vascularized organ printing [13–15].

Gelatin/siloxane hybrid inks represent a combination of natural and synthetic components for extrusion-based printing. Gelatin is a biocompatible and biodegradable polymer that can provide flexible hydrogel-like structures, while siloxane-based components may contribute to the structural stability of printed constructs [9]. These hybrid inks can be processed by different printing approaches, including extrusion, injection, and laser-based methods, which broadens their potential use across printing platforms. However, extrusion-based printing of gelatin/siloxane systems presents challenges associated with the interaction of process parameters such as composition, temperature, extrusion velocity, and pressure [11,15]. These parameters can affect filament formation, dimensional consistency, and interlayer adhesion in printed constructs [16,17].

Computational modeling, particularly finite element method (FEM)-based simulation, can support the analysis of extrusion behavior in gelatin/siloxane hybrid materials. FEM simulations allow the evaluation of ink flow behavior and the resulting filament response while considering the influence of composition, temperature, velocity, and pressure. By analyzing factors such as mechanical strength, flexibility, and surface quality, simulation can support the comparative screening of printing conditions and help interpret relationships between material properties, process parameters, and printed outcomes. Therefore, computational modeling can be used as a complementary tool for assessing extrusion-based printing behavior under defined experimental conditions [18–24].

Statistical analysis using a factorial design of experiments (DoE) enables the preprocessing and organization of experimental data for subsequent machine-learning analysis. In this study, a convolutional neural network (CNN) was trained using optical microscope images of printed meshes. Image processing was implemented to simplify the analysis and support the comparison of printed structures obtained under different printing conditions. In this context, machine-learning models can be used to classify printed mesh images and estimate image-derived morphological parameters, such as filament diameter and similarity to a CAD target, based on the available experimental dataset [25–27].

In this context, we present a computational workflow for screening key process parameters, including velocity, pressure, and temperature, for extrusion-based 3D printing of a gelatin/siloxane hybrid material. The workflow integrates rheological and mechanical characterization of the ink with FEM simulations in SolidWorks and an image-based ML classifier trained on printed mesh images. Within the tested parameter ranges, this approach was used to compare filament diameter and 2D similarity to a CAD target for printed meshes. The aim of the study is therefore to provide a comparative methodology for printability assessment under the evaluated conditions rather than to claim generalized optimization, material superiority, full 3D fidelity, or biological validation.

**A correction has been made to Section 2.2. Ink Characterization, Paragraph 1:**

The rheological properties of the gelatin/siloxane hybrid material (22G/78S) were characterized using a TA Instruments AR 2000ex rheometer (New Castle, DE, USA). Measurements were performed replicating the BIO X 3D printer’s operating parameters, including temperature, velocity, and pressure. Viscosity was analyzed in flow mode using a 25 mm diameter geometry. Compression resistance was computed at four stages: before and after printing, after crosslinking at the print bed temperature, and after UV radiation treatment. Tests were conducted using a TA Instruments Q800 DMA (New Castle, DE, USA) under controlled conditions (32 °C, vacuum atmosphere), with a force range of 0.001–18 N and a compression rate of 100 Pa/s. The material’s chemical composition was examined with a Thermo Nicolet 6700 FT-IR spectrophotometer (Madison, WI, USA) across three stages: before printing, crosslinking, and post-UV treatment. The analysis investigated the effects of physical and chemical crosslinking on chemical bonds and compression resistance. These analyses were used to characterize the structural and mechanical changes in the material before printing, after crosslinking, and after UV treatment under the evaluated processing conditions [3,5,9,11,16,28–30].

**A correction has been made to Section 2.3. Computational Model, Paragraph 1:**

The computational model for evaluating the extrusion behavior of the gelatin/siloxane hybrid material was developed using FEM in SolidWorks 2022 academic software. A volume-flow simulation based on the material’s physicochemical properties was conducted to estimate its thermomechanical response under the evaluated pressures, temperatures, and velocities within the BIO X 3D printer cartridge. The BIO X printer cartridge was designed to integrate the study parameters—temperature, velocity, and pressure—allowing analysis of material flow and deformation under defined conditions. A FEM-based simulation was developed in SolidWorks 2022 to evaluate the thermomechanical behavior of the gelatin/siloxane hybrid material during extrusion. A high-resolution finite element mesh was constructed, incorporating mechanical and fluid-dynamic parameters to represent the ink’s deformation and flow behavior. The simulated conditions included 200 kPa pressure, 45 mm/s velocity, and 32 °C temperature to analyze controlled deformation and support comparative screening of extrusion parameters.

**A correction has been made to Section 2.3.1. Mathematical Model for the Simulation of the Material and 3D Printing Process, Paragraphs 1–2:**

The mathematical model was used to describe the thermomechanical response of the 22G/78S gelatin/siloxane hybrid material through characterization tests, including thermogravimetric analysis (TGA), differential scanning calorimetry (DSC), and viscosity ramps as a function of temperature and shear stress performed on the rheometer [18,19,31–34].

The mathematical relationships were expressed using Astrid’s mathematical model, which was developed based on the flow rate equation, where extrusion velocity is calculated through FEM using specialized software. This calculation considers the geometry and physicochemical properties of the material. The flow rate (Q) is mathematically described as the product of extrusion velocity and the cross-sectional area of the nozzle, allowing estimation of volumetric flow behavior. Assuming maximum velocity equals extrusion velocity, a relationship with flow power was established. The Hagen–Poiseuille equation was then generalized and combined with the power-law equation. Finally, Equation (6) describes the estimation of filament diameter based on FEM calculations. This estimation considers the extrusion velocity and maximum velocity obtained from FEM simulations. Additionally, the elastic behavior of the ink was included because it may influence the material response during extrusion.

**A correction has been made to Section 2.3.2. Finite Element Method Computational Model of the 3D Printing Process, Paragraph 1:**

The thermomechanical properties of the gelatin/siloxane hybrid material, studied by Valenzuela-Reyes et al. [16], were input into SolidWorks to analyze compression resistance, shear stress, dynamic viscosity, and controlled deformation during extrusion-based printing. A fluid simulation incorporating 0–200 kPa compression stress, 5–45 mm/s horizontal velocity, and a constant temperature of 32 °C was used to study material deformation. A thermal barrier simulating a steady temperature of 10 °C in the print bed was used to evaluate its effects on filament properties, including shear stress. The software interface was also used to estimate the expected filament diameter considering the evaluated printing parameters and temperature-induced crosslinking conditions [18–24].

**A correction has been made to Section 2.3.3. Machine Learning Model, Paragraphs 1–2:**

The ML CNN classifier model was trained to analyze optical images of printed meshes captured using a Tomlov ×1000 optical microscope. The images were preprocessed by converting them to grayscale, binarizing them, applying a Gaussian filter, and resizing them using physical measurements obtained with a digital caliper. The dataset was split into training (80%) and validation (20%) sets. The images were labeled as “High” or “Low” according to their planar similarity to the CAD design, using metrics such as structural similarity (SSIM) and dimensional similarity (SD) (Figure 1a) [25–27,35].

Before being input into the ML model, the images were resized to a matrix size of 128 × 128. The dataset size was progressively expanded to 100 image batches, starting from 250 images. The hyperparameters were adjusted to improve training stability. Recall (R) was used as an evaluation metric. The model architecture was based on a convolutional neural network (CNN) designed to classify printed mesh images and compare them with the CAD design according to image-derived similarity criteria. This analysis assessed filament diameter and planar CAD similarity from optical images with dimensions of 128 × 128 pixels. The convolutional and pooling blocks were composed of filters of size 3 × 3, with depths of 32, 64, 128, and 256, and included batch normalization and MaxPooling2D to reduce dimensionality. Subsequently, a flattening layer was followed by a fully connected layer with 256 neurons and ReLU activation, together with L2 regularization to reduce overfitting. A dropout layer with a rate of 0.5 was used to further mitigate overfitting. The output layer was a single neuron with sigmoid activation, generating a binary value: 1 to classify “High” similarity and 0 to classify “Low” similarity with respect to the CAD design. This model analyzed images from the BIO X 3D printer to identify prints with higher image-derived similarity to the CAD target. The corresponding printing parameters were then retrieved using the predefined experimental matrix [25–27,35].

**The title of Section 2.4 has been corrected from “Validation 3D Printing Test” to “Printability Assessment Test”. A correction has been made to Section 2.4, Paragraph 1:**

The 3D printing assessment tests were conducted using 3 mL of ink composed of the gelatin/siloxane hybrid material (22G/78S), loaded into the cartridges of the BIO X 3D printer (CELLINK, Gothenburg, Sweden). A CAD design of a 10 mm × 10 mm mesh, as shown in Figure 1a, was printed using the parameters estimated from the ink characterization: applied pressure of 180 kPa, printing velocity of 25 mm/s, and temperature of 32 °C. The objective was to perform a statistical comparison between the experimentally obtained parameters and those estimated through FEM, based on length (L), width (W), and filament diameter (DF), and then estimate the planar CAD similarity (S). Images of the printed constructs were captured using an optical microscope (Tomlov ×1000), and these images were used to train the ML model, considering the printing parameters detailed in Table 1. Finally, the CAD design from Figure 2a and the printed mesh CAD design from Figure 2b were used to support filament diameter measurement and comparison among the three estimation methods: experimental, FEM simulation, and ML model [5,9,10,15,16].

**The subtitle “Manual Calculation of Percentage Similarity Between the Printed and the CAD Design” has been corrected to “Manual Calculation of 2D Percentage Similarity Between the Printed Mesh and the CAD Design”. A correction has been made to this subsection, Paragraphs 1–2:**

Printed meshes of 10 mm × 10 mm were manually measured using a digital vernier caliper to calculate the similarity percentage between the printed structure and the CAD design. This calculation allowed comparison between the trained machine-learning model and the physical prints using metrics such as MSE, MAE, and Pearson correlation coefficient to determine which estimation process showed closer agreement with the measured values.

The manual calculation was carried out using the following equations based on the measurements of the printed mesh: measured length (Lm), width (Wm), and the nominal CAD specifications: length (LCAD) and width (WCAD). The percentage error for each parameter was computed. Subsequently, a normalized average error was calculated, and the percentage similarity was obtained. This process quantified the dimensional deviation for each parameter and expressed the overall planar similarity as a percentage [25–27,35,36].

**A correction has been made to Section 2.5. DoE Study, Paragraph 1:**

Minitab software was used to conduct DoE analysis using a full factorial DoE with three factors and two, five, and five levels to compare experimental estimation, FEM simulation, and training of the ML CNN classifier model. In this study, the independent variables were nozzle size (A, G), applied pressure (B, kPa), and printing velocity (C, mm/s). The metrics for training the ML model were employed as response variables, as shown in Table 1. The purpose of this approach was to compare the experimental DoE results with the outputs obtained from FEM simulation and the trained ML classifier using correlation coefficients, linear regression equations, MSE, and MAE. This comparison was intended to assess agreement among the estimation methods and the physical measurements obtained [25–27,35,36].

**The title of Section 2.6 has been corrected from “Experimental Validation of DoE Regression Model and Machine Learning Model” to “Comparative Statistical Assessment of the DoE Regression and Machine-Learning Models”. A correction has been made to Section 2.6, Paragraph 1:**

The ML model was evaluated using statistical measures such as MSE and MAE, allowing comparison of the factorial DoE results, FEM simulation, and ML-based estimates. The MAE, representing the average absolute difference between predicted and actual values obtained in experimental tests, was determined using Equation (16). The MSE, derived from the residuals of the linear regression comparing actual values with predicted values, was calculated using Equation (17). The CNN classifier was evaluated by comparing linear regression statistics, MSE, and MAE. Additionally, Pearson correlation was conducted using Equation (18) to determine whether a linear relationship existed among the percentage similarity values obtained from the experimental tests, FEM simulation, and ML model. For the experimental tests and FEM simulation, pressure, temperature, and printing velocity were evaluated. For the CNN-based ML model, nozzle size, pressure, and speed were considered to compare average filament diameter and planar CAD similarity of the printed meshes [25–27,35,36].

**The title of Section 3.1 has been corrected from “Gelatin/Siloxane Ink Synthesis Experimental Validation” to “Gelatin/Siloxane Ink Synthesis and Characterization”. A correction has been made to Section 3.1, Paragraphs 1 and 3:**

The gelatin/siloxane hybrid material exhibited shear-thinning behavior, characterized by decreasing viscosity with increasing shear stress. The maximum viscosity of 3 × 10^5^ Pa·s occurred at 5 Pa, while the minimum viscosity of 0.089 Pa·s was observed at 50 Pa. These findings suggest a working pressure range for extrusion-based 3D printing using the BIO X 3D printer at a viscosity of 0.089 Pa·s, as shown in Figure 2a. Temperature also influenced viscosity, with the maximum recorded at 304.2 K and the minimum at 307.2 K, indicating shear-thinning behavior between 304.2 K and 307.2 K. Based on these results, a selected set of printing conditions for the gelatin/siloxane hybrid material was identified at 50 Pa and 307.2 K [16,28–30].

Before printing, mechanical compression resistance characterization revealed that constructs printed with the gelatin/siloxane hybrid material exhibited a resistance of 180 kPa and a deformation degree of 0.025%. Post-yield stress and deformation were observed with stable behavior, related to rheological and 3D printing tests (Figure 4). Following the 3D printing process, mechanical compression resistance of 183 kPa was obtained with a deformation degree of 0.027%. Similarly, deformation remained stable after the yield stress. This behavior could be attributed to the material characteristics relevant to extrusion-based printing of hydrogel-based mesh constructs (Figure 5) [9,16,28–30].

**A correction has been made to Section 3.2. Finite Element Method Simulation of the 3D Printing Process, Paragraph 1:**

A computational model for the 3D printing process was developed using a volume-flow simulation in SolidWorks 2022, incorporating the material’s physicochemical properties. The model analyzed the thermomechanical behavior of the material under varying pressures, temperatures, and velocities within the BIO X 3D printer cartridge (Figure 6). The simulation revealed the influence of printing parameters on material flow. The pressure and viscosity at the 25G nozzle (0.250 mm diameter) were determined, with ranges of 270–10 kPa for pressure and 10.38–0.250 Pa·s for viscosity. These results differ from experimental conditions, where a pressure of 170 kPa, a temperature of 31 °C, and a printing velocity of 25 mm/s were used. The model provided insights into the effects of printing parameters on material flow, identifying potential causes of nozzle obstruction. The relationships between shear rate and shear stress were examined to evaluate the shear-thinning behavior associated with stable extrusion under the tested conditions. Deviations from this behavior could result in inadequate flow or nozzle clogging. The Hagen–Poiseuille equation was used to estimate printing conditions for subsequent experimental comparison. After 70 simulations, a FEM-guided parameter set was identified: 171 kPa pressure, 31 °C temperature, and 0.026 m/s extrusion velocity. Shear stress in the nozzle geometry ranged from 63.24 Pa to 829.60 Pa, while dynamic viscosity averaged 2.88 Pa·s, remaining below the material’s maximum viscosity of 3 × 10^5^ Pa·s. This supported the use of the identified conditions for obtaining stable material flow and printed meshes with improved planar CAD similarity under the evaluated conditions. During printing, the filament formation of the 22G/78S material was observed at 10 °C. Viscosity increased from 3.66 Pa·s to 23.16 Pa·s, with shear stress rising from 50.6 Pa to 80 Pa, supporting filament formation. Upon contacting the print bed, the temperature dropped from 32 °C to 27 °C, aiding crosslinking and preserving filament properties (Figure 7). The estimated filament diameter of 0.253 mm closely matched the nozzle diameter of 0.250 mm (Figure 8). Despite slight swelling during crosslinking, controlled printing preserved dimensional and consistent mechanical properties. These findings support the use of the computational model as a screening tool to compare extrusion conditions and estimate filament formation under the evaluated parameter range [18–24,34,37,38].

**A correction has been made to Section 3.3. Estimation of Printing Parameters Using a Machine Learning Model, Paragraphs 1–** **4:**

A machine learning model was developed to classify printed mesh images and estimate image-derived similarity between the printed meshes and the CAD design. A dataset comprising physical measurements (length, width, and filament diameter) and estimated data, including the similarity percentage of the printed meshes measured both manually and using the SSIM index, was collected. The data were integrated into an Excel file, enabling image resizing and balancing of the “High” and “Low” labels through weight assignment and augmentation techniques, particularly for the manually labeled “High” images. Subsequently, a binary classifier based on a CNN was designed by splitting the dataset into 80% for training and 20% for testing. The training was carried out progressively, increasing the number of image batches of 100 and applying Early Stopping to reduce overfitting. With the addition of six extra batches, 850 images were reached, ensuring adequate training [25–27,36].

The hyperparameters were adjusted during the training stage, and metrics such as F1-score, accuracy, and recall were monitored. The classifier yielded AUC values of 0.87 for the “High” label and 0.82 for the “Low” label, indicating its ability to distinguish between the image-based similarity classes defined in this dataset. The overall AUC value was calculated as 0.97 for the image-classification task. Separately, MSE and MAE were calculated to evaluate agreement between estimated and measured dimensional or similarity-related values. These metrics describe different aspects of model performance and should not be interpreted as directly equivalent to regression-based R2 values [25–27,36].

The binary classifier selected Figure 9, which exhibited the highest image-derived similarity relative to the CAD design: 94.133% according to the SSIM-based calculation and 97% using the manual 2D similarity calculation. Additionally, a filament diameter of 0.252 mm was recorded (Figure 10) [25–27,36]. 

The results indicate that the CNN-based classifier can be used to classify images of printed meshes and identify samples with higher image-derived similarity to the CAD design within the evaluated dataset. Therefore, the model should be interpreted as a complementary screening tool for comparing printing conditions rather than as a method for determining universally applicable printing parameters.

**The title of Section 3.4 has been corrected from “Validation 3D Printing Test” to “Comparative 3D Printing Test”. A correction has been made to Section 3.4, Paragraph 1:**

In this study, a total of 850 meshes (10 mm × 10 mm) were fabricated following the experimental matrix defined by the factorial Design of Experiments (DoE) framework. The printed meshes exhibited dimensional variability, with lengths ranging from 7.3 mm to 15.2 mm (mean: 11.028 ± 1.3 mm), widths spanning 8.5 mm to 14.8 mm (mean: 11.044 ± 1.2 mm), and filament diameters between 0.21 mm and 5 mm (mean: 1.098 ± 0.9 mm) (Figure 11). Each mesh was measured using digital calipers, and optical images were captured for statistical analysis and ML model training. The 2D similarity percentage between the printed mesh images and the CAD reference varied from 0% to 97% (mean: 54.5%). Furthermore, the experimentally obtained printing parameters were systematically compared with those derived from FEM simulation and ML model-based image analysis, providing a comparative assessment of the experimental measurements, FEM-based estimates, and ML-based image analysis [25–27,36].

**A correction has been made to Section 3.5.2. Response Surface Design, Paragraphs 2–3:**

The response surface plots for filament diameter and similarity percentage were generated after filtering out non-significant factors and assigning the nozzle as the x-axis variable, as it had the most statistically significant effect. Note that the Pressure*Velocity interaction was discarded from the surface analysis due to its lack of significance. The filament diameter surface plot (Figure 14) demonstrated that, in the Nozzle*Pressure analysis, use of the 27G nozzle produced a smaller filament diameter under the evaluated pressure range. However, since the diameter decreased excessively at higher pressures, the selected condition associated with smaller filament diameter in this analysis was 170 kPa with a 27G nozzle. Similarly, the Nozzle*Speed analysis revealed that the least favorable condition, with a diameter exceeding 5 mm, occurred with a 25G nozzle at 5 mm/s, while the filament diameter decreased proportionally with increasing print speed and when using the 27G nozzle. Therefore, the condition associated with an expected diameter of less than 1 mm corresponded to a 27G nozzle, 25 mm/s velocity, and 170 kPa pressure [18,25–27,31,35–38].

The response surface plot for SSIM (Figure 15) calculated by the ML model showed an average value in the study range of approximately 50–60%. For the Nozzle*Pressure interaction, a pressure between 170 and 180 kPa and use of a 25G nozzle produced higher SSIM values within the evaluated dataset, while high pressure and use of the 27G nozzle were associated with lower the similarity percentage relative to the CAD design. Furthermore, the Nozzle*Velocity interaction indicated that both 25 mm/s with a 27G nozzle and 5 mm/s with a 25G nozzle reduced the SSIM value; therefore, the conditions associated with higher SSIM values in this dataset were approximately 15–20 mm/s with a 25–27G nozzle, with SSIM further increasing at approximately 180 kPa, 15 mm/s, and a 25–27G nozzle. Using the ML model to calculate SSIM allowed comparison of the image-derived similarity among printed meshes, supporting the identification of parameter sets associated with higher planar CAD similarity. This integrated approach takes into account the physicochemical properties of the ink and the final structure of the printed mesh [9,18,25–27,31,35–38].

**A correction has been made to Section 3.6. Statistical Comparison Between the Experimental Estimation, FEM Simulation, and ML Model, Paragraphs 2–** **4:**

The initial physicochemical characterization tests allowed an experimental estimation of the printing parameters. Likewise, simulation of the physicochemical properties of the 22G/78S ink through volumetric fluid simulation using FEM facilitated the estimation of printing parameters (Table 2). The printing assessment tests enabled comparison of different meshes within the previously defined intervals, which allowed assessment of the printing parameters estimated both experimentally and via FEM simulation [18,25–27,31,35–38]. Subsequently, the printed meshes were measured using a digital caliper to corroborate the FEM simulation estimates. Images captured with the Tomlov digital microscope were used to train the binary CNN classifier, which determined the similarity percentage concerning the CAD manually and using SSIM (Table 2). Finally, by considering the printing parameters and comparing the factorial DoE experiment matrix, the printing parameters were estimated by selecting the image that exhibited the highest image-derived similarity to the CAD design (Table 2) [9,18,21,25–27,31,35–38].

Through statistical analysis of the factorial DoE, the experimental setting yielded a filament diameter of 1.098 mm and a CAD similarity of 54.5% for the evaluated mesh images. The FEM-based setting yielded an average filament diameter of 0.253 mm and a similarity of 92.35%, while the ML-based image screening identified printed meshes with an average filament diameter of 0.252 mm and a maximum image-derived similarity of 94.13% under the tested conditions. These values, shown in Table 3, should be interpreted within the scope of the metrics used in each method. In particular, the AUC value reported for the CNN classifier and the R2 values obtained from the regression analyses describe different modeling tasks and therefore are not directly comparable as a single ranking criterion. Accordingly, the results support the use of FEM and image-based ML as complementary tools for screening printing conditions and comparing planar CAD similarity in this dataset, rather than establishing an absolute hierarchy of model performance.

The Pearson correlation analysis showed a positive relationship between the manual 2D similarity calculation and the similarity class learned by the binary CNN classifier, with a correlation coefficient of 0.841. This result suggests that the classifier captured relevant image-derived differences associated with the predefined similarity categories. A negative correlation was observed between filament diameter and CAD similarity, with a correlation coefficient of −0.316, indicating that larger filament diameters were generally associated with lower planar similarity percentages in the evaluated meshes. Based on the combined FEM and ML screening, the parameter set consisting of a 25G nozzle, 170 kPa pressure, 25 mm/s printing speed, and 32 °C temperature was associated with an average filament diameter of 0.253 mm and a CAD similarity of 92.35% under the tested conditions. These findings should be interpreted as comparative results within this gelatin/siloxane dataset, not as evidence of universal optimization.

**A correction has been made to Table 3:**

The column previously labeled “AUC/R^2^” has been revised to avoid implying that AUC and R^2^ are directly comparable performance metrics. The table now separates the metrics according to the corresponding modeling task, using “AUC for CNN classification” for the binary image-classification model and “R^2^ for regression-based comparison” for the DoE/FEM-related regression analyses. Entries not applicable to a given model type are indicated as “N/A”. This revision clarifies that AUC and R^2^ describe different aspects of model performance and should not be interpreted as equivalent ranking criteria.

**Table 3:** Statistical comparison of the estimation methods for the printing parameters of the gelatin/siloxane ink.

Estimation Methods Comparison						
Estimation Methods	Filament Diameter (mm)	Similarity Percentage (%)	AUC for CNN Classification	R^2^ for Regression-Based Comparison	MSE	MAE
Experimental	1.098	54.5	N/A	0.79	0.9005	1.086
FEM simulation	0.253	92.35	N/A	0.85	0.0034	0.004
ML model	0.252	94.13	0.97	N/A	0.0031	0.003

**A correction has been made to Section 4. Discussion, Paragraphs 1–6:**

The experimental assessment of the printing conditions for the gelatin/siloxane hybrid material focused on rheological characterization and mechanical testing. The relationship between viscosity and shear stress was analyzed, showing that a shear stress of 50 Pa corresponded to an applied pressure above 150 kPa. Shear-thinning behavior suitable for extrusion was observed between 30 °C and 32 °C, with viscosity increasing toward 10 Pa·s at higher temperatures. Consequently, the printing temperature was set at 32 °C. The BIO X 3D printer velocity was selected using parameters from previous studies, supporting comparison between the experimental measurements and the computational model [9,18,25–27,31,35–38].

Mechanical testing showed a slight decrease in compression strength after printing, while UV treatment increased the measured resistance under the evaluated conditions. FTIR analysis associated these changes with silanol oxidation and gelatin–siloxane interactions, including conversion of Si–OH to Si–O and changes in the intensity of functional groups. These physicochemical changes may contribute to the structural stability of the printed material and to stable extrusion behavior under the tested conditions. The material was printed at 180 kPa and 32 °C, reaching 50–90% image-derived filament similarity as measured by optical microscopy [9,18–24,36–38].

Based on Astrid’s mathematical model implemented in SolidWorks 2022, the computational model used thermomechanical properties such as density, specific heat, thermal conductivity, and viscosity. The Hagen–Poiseuille equation was used to estimate extrusion velocity and to identify parameter sets associated with stable material flow. Simulations revealed dynamic viscosity variations from 10 Pa·s to 0.025 Pa·s, indicating shear-thinning behavior under the evaluated conditions. After 70 simulations, a FEM-guided parameter set of 170 kPa pressure, 31.2 °C temperature, and 25 mm/s printing velocity was obtained [9,18–27,31,35–38].

The training of the binary CNN classifier enabled the identification of printed mesh images with higher similarity to the CAD design and supported the calculation of image-derived similarity percentages by comparing the experimental, FEM-based, and ML-based estimates. It is important to note that the ML model considers morphological and structural features from 2D optical images of the printed constructs and does not directly evaluate mechanical performance, biological behavior, or general superiority across different materials or printing systems. In contrast, the FEM simulation was used to estimate material flow and filament formation under the evaluated extrusion conditions. Therefore, the combination of FEM and image-based ML should be interpreted as a complementary screening strategy for comparing printing conditions and planar CAD similarity, considering the physicochemical properties of the ink and the final structure of the printed meshes [9,18–27,31,35–38].

The ML model calculated the similarity percentage of the printed mesh images, while the FEM simulation provided an estimate of filament diameter and printing response based on the mechanical and physicochemical properties of the ink. In the gelatin/siloxane hybrid material, printing parameters of a 25G nozzle, 170 kPa pressure, 25 mm/s printing velocity, and 32 °C temperature were associated with a CAD similarity of 92.35% under the evaluated conditions. Although this percentage was lower than the highest similarity value suggested by the ML-based image analysis, the slightly smaller filament diameter obtained for the printed mesh could affect layer stacking and structural integrity when additional layers are printed. These findings should, therefore, be interpreted as comparative results for planar printability and image-derived CAD similarity, rather than as direct evidence of full 3D fidelity or biological performance [9,18–27,31,35–38].

The factorial DoE enabled the comparison of printing-condition effects using polynomial regression models, response surface analyses, and response variables such as filament diameter and similarity percentage. This approach supported the comparison of parameter-estimation methods and the identification of printing conditions associated with higher planar CAD similarity based on 2D images captured with the Tomlov digital microscope. Additionally, variations among factors associated with the extrusion printing process using the BIO X printer were analyzed to identify parameters related to favorable structural and physicochemical responses under the evaluated conditions. However, further three-dimensional geometric validation and biological testing would be required before extending these findings to full scaffold fidelity, functional performance, or biomedical applications.

**A correction has been made to Section 5. Conclusions, Paragraph 1:**

This study supports the use of a computationally assisted workflow to evaluate extrusion printing conditions for a gelatin/siloxane hybrid material under the tested conditions. Rheological characterization, FEM-based flow simulation, and image-based analysis provided a practical framework for comparing filament diameter and 2D CAD similarity of printed meshes. Within this dataset, the FEM-guided parameter set was associated with filament diameters closer to the nominal nozzle dimension than the initial experimental setting, while the image-based ML analysis was useful for identifying printed meshes with higher planar similarity to the CAD target.

The oxidation of dispersed siloxane particles influenced the thermomechanical and rheological behavior of the gelatin/siloxane hybrid material, affecting its extrusion response and printed mesh formation. Statistical comparisons using factorial DoE, FEM simulation, and ML-based image analysis showed that the computationally assisted approaches can support the comparative screening of printing conditions. However, the reported similarity values are derived from 2D image-based metrics and should not be interpreted as direct evidence of full 3D fidelity, generalized process optimization, material superiority, or biological performance.

Therefore, these findings should be interpreted as evidence for comparative printability assessment within this gelatin/siloxane system rather than as proof of universal optimization or biomedical functionality. Further work should include three-dimensional geometric validation, mechanical assessment under application-relevant conditions, and biological testing before extending the conclusions to scaffold function or biomedical applications.

**A correction has been made to the correspondence contact information.**

The authors state that the scientific conclusions are unaffected. This correction was approved by the Academic Editor. The original publication has also been updated.
